# Supplementary material for: Multiscale Simulations of Membrane Adhesion Mediated by CD47-SIRPα Complexes
Source: J Chem Theory Comput. 2025 Feb 17;21(4):2030–42. doi: 10.1021/acs.jctc.4c01337 (PMC11866742; doi:10.1021/acs.jctc.4c01337)
Supplement: Supplementary file 1 — ct4c01337_si_001.pdf [file ct4c01337_si_001.pdf]

Supporting Information

Multi-scale simulations of membrane adhesion mediated by CD47-SIRPα complexes

Ruihan Hou[§,1,2], Shuanglong Ren[§,1], Rong Wang[2], Bartosz Różycki[*,3], Jinglei Hu[*,1,2]
[1]Kuang Yaming Honors School, Nanjing University, Nanjing 210023, China
[2]Department of Polymer Science and Engineering, Key Laboratory of High Performance Polymer Material and Technology of Ministry of Education, School of Chemistry and Chemical Engineering, Nanjing University, Nanjing 210023, China
[3]Institute of Physics, Polish Academy of Sciences, Al. Lotników 32/46, 02-668 Warsaw, Poland

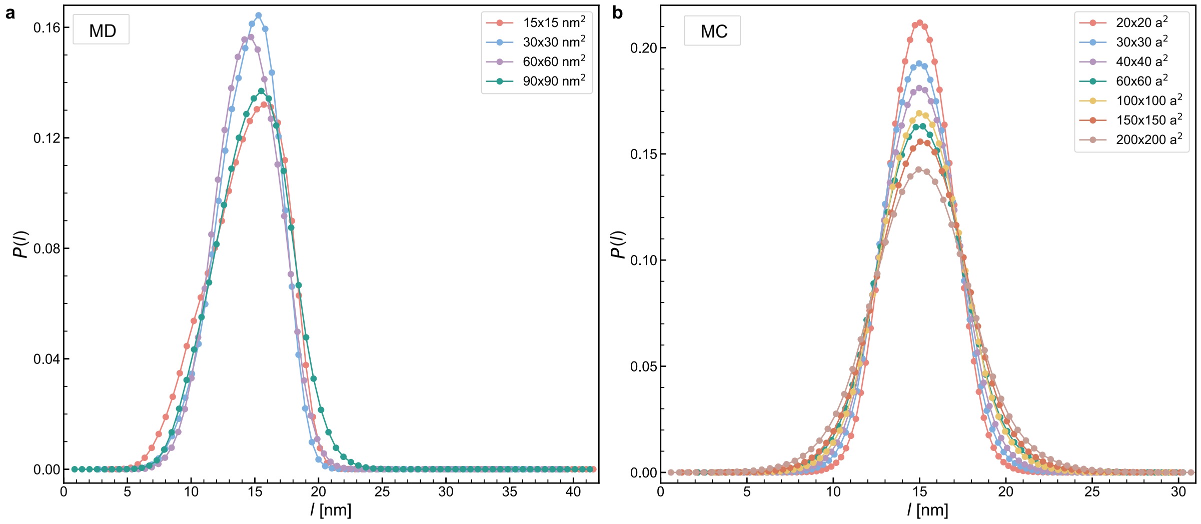

Figure S1: Distributions of the local membrane separation, $P(l)$, obtained from the coarse-grained MD (a) and MC (b) simulations with different membrane areas as specified in the legend.

[§]These authors contributed equally to this work.
[*]Corresponding authors: Bartosz Różycki (rozycki@ifpan.edu.pl) and Jinglei Hu (hujinglei@nju.edu.cn)

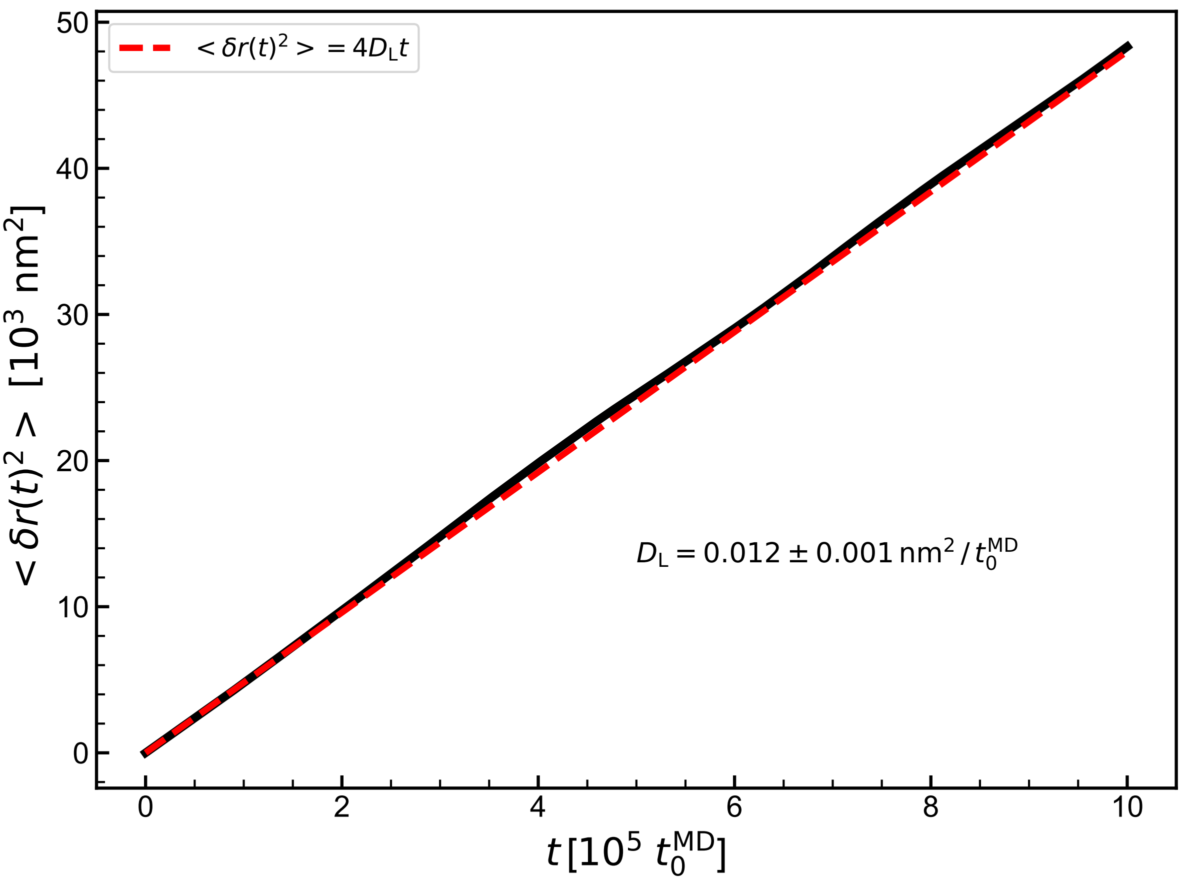

Figure S2: Mean squared displacement of the lipids as a function of time. The red dashed line corresponds the least-square fit of the MD data to the Einstein relation $\langle \delta r(t)^2 \rangle = 4D_{\mathrm{L}}t$, leading to the diffusion coefficient $D_{\mathrm{L}} = 0.012 \pm 0.001\, \mathrm{nm}^2/t_0^{\mathrm{MD}}$.
